# Multispectral PCCT and CBCT imaging for high precision radiotherapy through translation of imaging parameters with machine learning validation

**DOI:** 10.1038/s41598-025-33888-6

**Published:** 2026-01-08

**Authors:** Constantin Dreher, Abhinay Vellala, Victor Siefert, Florian Haag, Stefan Sawall, Jens Fleckenstein, Sven Clausen, Judit Boda-Heggemann, Stefan O. Schoenberg, Frank A. Giordano, Matthias Froelich

**Affiliations:** 1 Department of Radiation Oncology, University Medical Centre Mannheim, Medical Faculty Mannheim, University of Heidelberg, Theodor-Kutzer Ufer 1-3, 68167 Mannheim, Germany; 2 DKFZ-Hector Cancer Institute, University Medical Centre Mannheim, Mannheim, Germany; 3 Department of Radiology and Nuclear Medicine, University Medical Centre Mannheim, Medical Faculty Mannheim, University of Heidelberg, Mannheim, Germany; 4 Division of X-Ray Imaging and CT, German Cancer Research Center (DKFZ), Heidelberg, Germany; 5 Mannheim Institute for Intelligent Systems in Medicine (MIiSM), Medical Faculty Mannheim, University of Heidelberg, Mannheim, Germany; 6 Junior Research Group “Intelligent Imaging for adaptive Radiotherapy”, Mannheim Institute for Intelligent Systems in Medicine (MIiSM), Medical Faculty Mannheim, University of Heidelberg, Mannheim, Germany

**Keywords:** PCCT, HyperSight, Ethos, Multispectral imaging, IGRT, High-precision radiotherapy, Diagnostic markers, Medical research

## Abstract

Photon-counting CT (PCCT) is the mainstay of multi-spectral imaging, enabling quantitative tissue characterization. In radiation oncology, cone-beam CT is used daily for image-guided and online-adaptive radiotherapy. The novel HyperSight cone-beam CT imaging mode (CBCT), with enhanced image quality due in part to its enlarged detector size and optimized reconstruction modes, further facilitates quantitative image monitoring and high-precision radiotherapy. Integrating spectral PCCT information may further amplify its potential. Therefore, this study investigates whether qualitative and spectral quantitative PCCT-parameters can be translated to CBCT. An inorganic tissue-equivalent anthropomorphic phantom analysis was conducted using CBCT (iCBCT/iCBCT Acuros reconstruction, Pelvis/Pelvis Large preset) and PCCT (T3D (polychromatic reconstruction) with virtual monochromatic imaging (VMI)). Twenty regions with different CT numbers were assessed qualitatively and quantitatively. Image quality was highest for T3D PCCT. Quantitative analysis showed stronger agreement between CBCT (iCBCT Acuros) and PCCT-derived 60 and 67 keV VMI (concordance correlation coefficient (CCC) ≥ 0.595), compared to T3D (CCC ≤ 0.183), with CCC values significantly affected by CBCT presets and reconstruction method (*p* ≤ 0.001). Machine learning-based hierarchical clustering confirmed alignment between CBCT and PCCT-based VMI, but not T3D. This successful translatability of specific VMI levels paves the way for the integration of multi-spectral imaging into high-precision CBCT-based radiotherapy using PCCT.

## Introduction

Computed tomography (CT) imaging is a fundamental part of diagnostic imaging in oncologic radiology, serving not only for staging but also for treatment planning in radiation oncology. To date, the mainstream approach to simulation CT scans for treatment planning is based on energy-integrating detector (EID) CT examinations. However, diagnostic CT scans have evolved significantly over the past decades, with advancements in both image quality and quantitative oncologic imaging. With the approval of photon-counting CT (PCCT) scanners for daily clinical practice, a fundamentally improved CT-based diagnostic imaging modality has become available, offering enhancements over both traditional EID CT and monoenergetic reconstructions derived from classical dual-energy approaches^1^.

While standard EID diagnostic CT scans are routinely performed for oncological assessments and offer a robust radiologic marker for response prediction^2–4^, the novel PCCT technology offers the potential for even better diagnostic accuracy and tissue differentiation due to higher image resolution. This improvement may provide information about the tumor tissue that exceeds the capabilities of standard EID CT imaging^5,6^.

Simultaneously, imaging in radiation oncology has also advanced substantially over time. Treatment planning currently relies on simulation CT scans performed with EID CT scanners, which provide diagnostic-quality imaging. Image-guided radiotherapy (IGRT), the standard of care in daily clinical practice^7–10^, is generally based on repetitive cone-beam CT (CBCT) imaging for patient positioning and target localization prior to radiation. Further, the introduction of the advanced CBCT-based adaptive radiotherapy (ART) approach which allows for highly precise irradiation with online adjustments based on fractional imaging, has further increased the importance and application of CBCT imaging. However, CBCT scans at standard linear accelerators offer significantly lower image quality compared to diagnostic CT scans. This limitation restricts CBCT to robust radiation positioning without diagnostic utility^11,12^. Due to imaging artifacts and elevated noise levels, standard CBCT has not been widely adopted for further imaging analysis, either qualitatively or quantitatively. Its diagnostic potential is significantly limited, as demonstrated by studies investigating CBCT image quality^13,14^ and the conflicting results regarding reliability of quantitative radiomic parameter assessments^15–17^.

If the repetitive CBCT imaging performed during radiotherapy could achieve higher imaging quality, CBCT-based monitoring might offer significant additional information. This could include not only improving the visualization of cancer and organs at risk but also providing predictive markers of tumor response and normal tissue reactions. With the introduction of the novel HyperSight-CBCT imaging system (Varian, Siemens Healthineers), a high-quality CBCT modality featuring a combination of improved hardware and software characteristics (e.g., larger field of view, faster acquisition time with less motion artifacts, and optimized reconstruction modes) has become feasible for daily practice in radiation oncology, especially in case of online ART at the Ethos linear accelerator^18–21^. Initial clinical applications of this CBCT imaging mode have already demonstrated notable improvements in image quality and robustness of quantitative imaging parameters^20,22^.

The advantages of improved image quality and CT number stability in CBCT imaging could be further enhanced when combined with the spectral information provided by PCCT scans. Therefore, the objective of this study is to establish a translational framework in which advanced PCCT provides quantitative imaging parameters that serve as a reference for CBCT during radiation therapy. The present study constitutes a preliminary investigation, the objective of which is to assess the correlation and convergence of qualitative and spectral quantitative parameters between PCCT and advanced CBCT imaging. The results of this investigation may serve to lay the groundwork for a synergistic imaging approach that could support longitudinal quantitative image-guided monitoring and, ultimately, adaptive treatment.

## Methods

### Study concept and Phantom

**Fig. 1:**
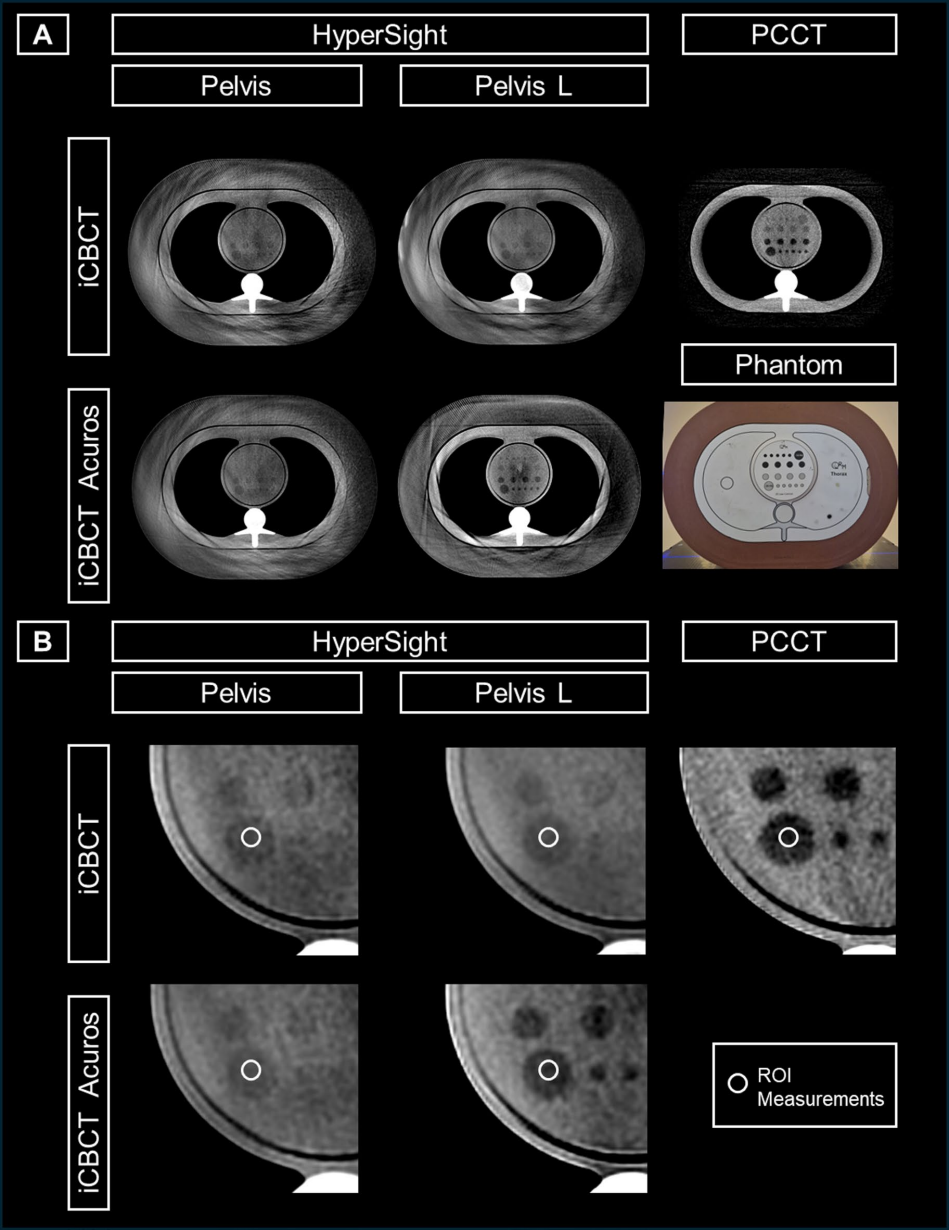
Inorganic tissue-equivalent anthropomorphic phantom design for image quality reading and CT number measurements with exemplified Regions of Interest (ROIs) being placed in one of the 20 different computed tomography (CT) number regions. Imaging with both HyperSight conebeam CT (CBCT) (preset Pelvis and Pelvis Large (Pelvis L) for the two reconstruction modes iCBCT and iCBCT Acuros) and photon-counting CT (PCCT) using T3D reconstruction with Q40. The WL/WW of the demonstrated slices are set to 60/230 and 80/250 for the preset Pelvis, and 65/200 and 35/105 for Pelvis Large (for the two reconstruction modes, iCBCT and iCBCT Acuros, respectively). The WL/WW is set to 40/80 for PCCT. The slice thickness is set to 3 cm. The overall view of PCCT/CBCT imaging is shown in axial orientation at the same height in the phantom (**A**), with a zoomed-in visualization of an exemplary ROI (**B**).

All experiments were performed using a semi-anthropomorphic thorax phantom (2D-Low-Contrast, Thorax, QRM, A PTW Company, Möhrendorf, Germany). The intersection lengths through the phantom (axial dimensions of the phantom: 40 × 30 cm) resemble ones observed in actual adult patients and thus represent a realistic clinical scenario. The length of the superior-inferior axis is 20 cm for the outer part of the phantom and 10 cm for the inner part. This phantom was equipped with a dedicated insert showing low contrast structures characterized by 20 dedicated tube-shaped regions (range: 5–15 mm in diameter), each with different CT-values in a range of -10 Hounsfield Units (HU) to -20 HU below the background of the phantom (see Fig. 1).

Measurements were performed using the PCCT scanner (NAEOTOM Alpha, VB20, Siemens Healthineers, Forchheim, Germany) and the O-ring linear accelerator Ethos (Varian, Siemens Healthineers) equipped with the novel HyperSight-CBCT scanning mode.

All methods were carried out in accordance with relevant guidelines and regulations. No animal- or human-related data are included in this analysis. The authors and their departments are the source of the data involved in this study. No external database was used.

### CT examinations

The clinical CT-based examinations of the phantom were performed using scan parameters comparable to those employed in daily clinical practice.

PCCT scans on the NAEOTOM Alpha were performed with standard polyenergetic mode. The scan characteristics were as follows:

- 120 kV, constant 250 mAs, QuantumPlus scan mode with two energy bins, Q40 reconstruction kernel, QIR-level: 3, 13.6 mGy CTDIvol (volumetric computed tomography dose index; 32 cm body dosimetry phantom).
- Data were reconstructed to a conventional CT image encompassing all measured photons (referred to as T3D at the scanner with polychromatic reconstruction generated from all detected x-ray photons above the lowest energy treshold, corresponding to the conventional image type in energy-integrating detector CT) and to virtual monochromatic images (VMIs) using spectral information from two energy bins^23^.

CBCT scans on the HyperSight-equipped Ethos (Varian Medical Systems Inc.), which will be named “CBCT” in the following sections of Material and Methods and Results, were performed with fully open collimation in two scans with two reconstruction modes “iCBCT” (_i) and “iCBCT Acuros” (_iA):

- The iCBCT mode utilizes an iterative reconstruction algorithm for noise reduction, while iCBCT Acuros is additionally characterized by an advanced scatter correction and increased CT-number accuracy^24–26^.

The following presets for the CBCT scans were selected because they enable a relatively high soft-tissue contrast due to the higher mAs:

- Preset “Pelvis”, 125 kV, 470.35 mAs (CBCT-Pel), 1.15 mAs per projection (80 mA * 14.3ms), 8.9 mGy CDTIvol (32 cm body dosimetry phantom).
- Preset “Pelvis Large”, 140 kV, 527.71 mAs (CBCT-PelL), 1.29 mAs per projection (90 mA * 14.3 ms), 13.6 mGy CTDIvol (for 32 cm body dosimetry phantom).

The default scan diameter (the field of view in the axial plane) is 53.8 cm and the maximum scan length is 24.1 cm. The voxel size is 1.05 mm in the x- and y-directions, with 2 mm slice distance in the longitudinal direction. A total of 409 projections are acquired during a scan time of 5.9 s for each protocol preset. The tube is operated continously, with the beam constantly on during the scan time. Filtration includes a bowtie filter, and a prefiltration of 1 mm Titanium, on top of the 1 mm AI inherent tube filtration.

### Assessment of image quality

Subjective evaluation of image quality was performed using a 5-point Likert scale^27^ on a representative axial slice taken from the middle of the phantom with 3 cm slice thickness for visualization. The 20 regions with different CT numbers within the phantom were rated by two readers as follows:

- 1 = no regions’ boundaries identifiable, totally obscured.
- 2 = questionable recognition of the regions’ boundaries, marked artifacts.
- 3 = recognition of the regions’ boundaries with moderate confidence.
- 4 = recognition of the regions’ boundaries with high confidence.
- 5 = recognition of the regions’ boundaries with excellent confidence.

The evaluation was conducted for the four CBCT scans, the PCCT T3D reconstruction, and the derived VMI datasets from 40 to 180 keV in 20 keV steps. Individual changes at the CT window-level were allowed.

The assessments were performed by two readers:

- R1: radiologist with 11 years of experience.
- R2: radiation oncologist with 8 years of experience in CT and CBCT imaging, respectively.

### Assessment of quantitative CT parameters

Separate regions of interest (ROIs) were placed at the center of each of the 20 dedicated CT-number regions (ROI area: 5-6mm^2^) across the five CT/CBCT scans. The means of CT numbers within the ROIs were extracted as quantitative parameters of the five CT scans and the VMI (from 40 keV to 190 keV in 1 keV steps). The delineation of the ROIs was conducted by both readers in consensus (Fig. 1).

### Statistical analysis

Statistical analysis was performed using Python (v3.13) and SPSS software package (v28, SPSS; Chicago, IL, USA).

Python packages included *pandas v1.15* (data manipulation), *numpy v.1.24*,* scipy* (statistics) and *matplotlib*,* seaborn v0.12* (visualization). To quantify the agreement between CT numbers from different imaging modalities, we computed the concordance correlation coefficient (CCC) for all possible combinations of scanner modes. The results were organized into a symmetric matrix. We visualized this matrix using a heatmap to illustrate global concordance patterns. Additionally, bar plots were used to compare the CCC of each modality with that of the selected reference scanner modes. We applied the machine learning approach of agglomerative hierarchical clustering based on the Python package *sklearn* to investigate similarity across energy-dependent imaging features. First, we normalized the feature matrix using Min–Max scaling to the range [0, 1] to ensure equal weight across features. Clustering was performed using the complete linkage method and the Euclidean distance metric. We constructed the resulting dendrogram using the *SciPy v1.11* package *scipy.cluster.hierarchy*. To visualize the clustering structure in reduced dimensions, we performed principal component analysis (PCA) on the normalized feature matrix. This analysis provides an interpretable 2D representation of high-dimensional similarity patterns that complements the hierarchical clustering dendrogram. PCA was applied using the *scikit-learn* library, with the number of components fixed at two (2D projection) for graphical visualization. The PCA algorithm performed an orthogonal transformation so that the new variables (principal components) maximize the variance captured from the original dataset. The input data (all imaging features, excluding sample labels) was scaled using Min–Max normalization to the range [0,1].

Inter-reader reliability was tested by weighted Cohen’s Kappa test (K). Significant differences of image quality readings and CCCs were tested by Friedman’s test with post hoc Bonferroni adjustment. A p-value of 0.05 was considered statistically significant.

## Results

### Image quality analysis of CBCT and PCCT

#### Inter-reader reliability of image quality assessment

Inter-reader reliability of image quality was good in pooled analysis of all scans (K = 0.574), moderate for CBCT-PelL_i (K = 0.496), fair for T3D (K = 0.389), fair for CBCT-Pel_i (K = 0.381), good for CBCT-Pel_iA (K = 0.508), and good for CBCT-PelL_iA (K = 0.500).

Inter-reader reliability of image quality ranged from good to high for PCCT-derived virtual monoenergetic images at 40, 60, 80, 100, 120, 140, 160, and 180 keV (K = 0.765, 0.489, 0.500, 0.660, 0.517, 0.524, 0.524, and 0.524, respectively).

### Comparison of image quality between PCCT and CBCT scans

**Fig. 2:**
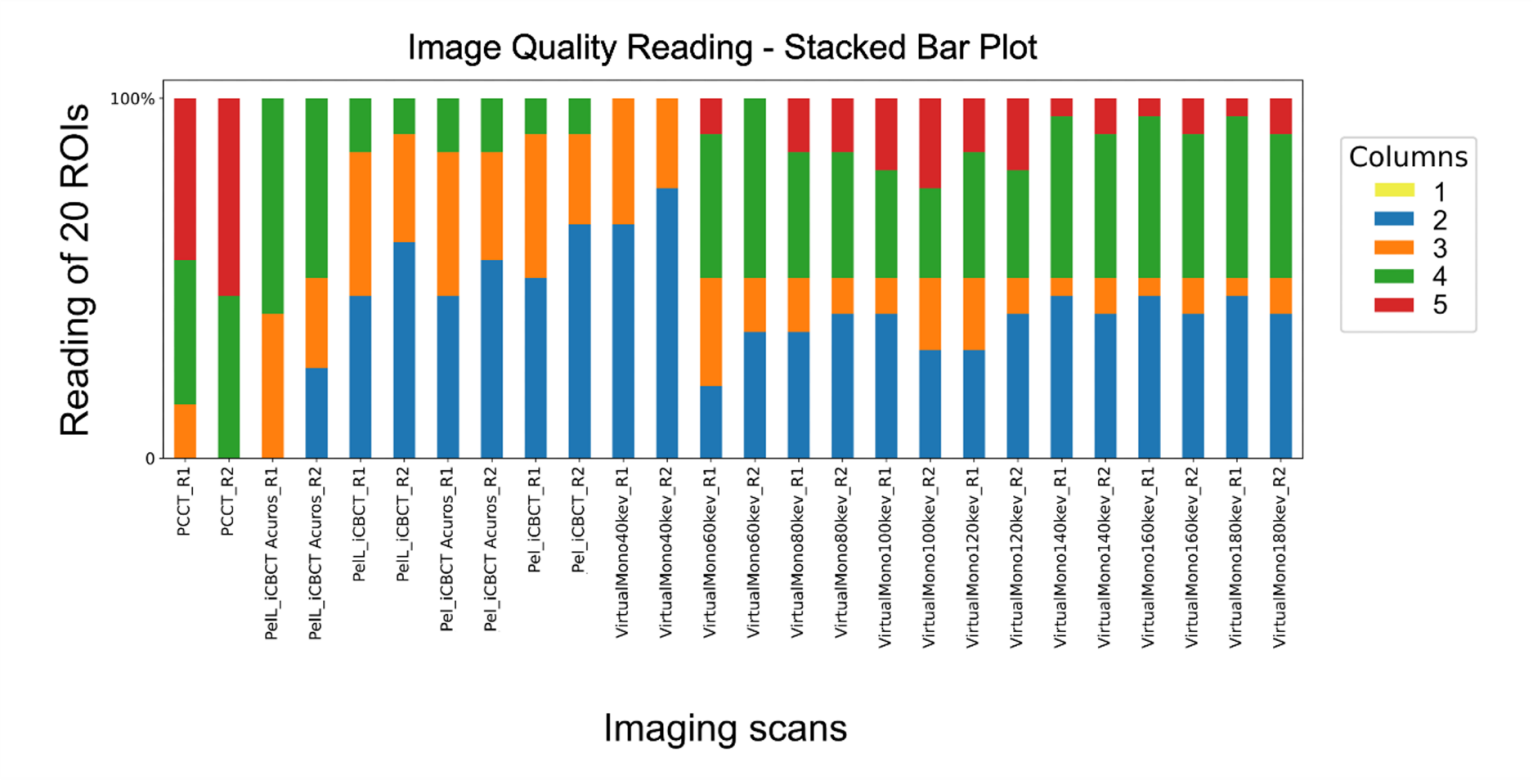
Stacked Bar Plot of image quality reading of an inorganic tissue-equivalent anthropomorphic phantom scan with HyperSight conebeam computed tomography (CBCT) (preset “Pelvis” (Pel), and “Pelvis Large” (PelL) with the reconstruction modes “iCBCT” and “iCBCT Acuros”) and with photon-counting CT based T3D (PCCT), and virtual monoenergetic reconstructions for 40–180 keV in 20 keV steps (VirtualMono40–180 keV). Reading according to a Likert-Scale with 1–5 points (columns 1–5) by two readers (R1 and R2) with possible windowing allowed in all case. As indicated in the Likert-Scale only values of 2–5 were assigned. Image quality of PCCT is characterized by higher reading values compared to CBCT and the virtual monoenergetic reconstructions are characterized by overlapping values compared to CBCT, and decreased values compared to PCCT.

Image quality readings from both readers demonstrated nominally decreasing values from PCCT to CBCT_iA and CBCT_i reconstruction, with nominally higher values for PelL compared to Pel (Fig. 2).

In the comparison of the five polyenergetic scans, image quality was significantly different for R1 (*p* < 0.001) and R2 (*p* < 0.001), but in post hoc analysis image quality of T3D was significantly increased compared to CBCT-Pel_i/PelL_i/Pel_iA (p_adj_<0.001) but not compared to CBCT-PelL_iA for R1 (p_adj_=0.801). For R2 the rating of T3D was significantly increased compared to each of the four CBCT presets (p_adj_<0.019).

In the analysis for significant differences among the five imaging polyenergetic CT/CBCT scans and the VMI 40/60/80/100/120/140/160/180 keV of PCCT, the rating values of the VMI were only significantly different with decreased values for VMI 40 keV compared to CBCT-PelL_iA (p_adj_≤0.001 for R1 and p_adj_=0.044 for R2) and increased values for VMI 100 keV compared to CBCT-Pel_i/PelL_i for R2 (p_adj_=0.016/0.032). In the comparison of VMI with T3D, the nominally increased image quality readings of T3D were significantly different compared to VMI at 40/100/120/140/160/180 keV for R1 (p_adj_≤0.047) and at 40/60/80/120/140/160/180 keV for R2 (p_adj_≤0.012).

### Correlation of the CT number measurements by CBCT and PCCT

**Fig. 3:**
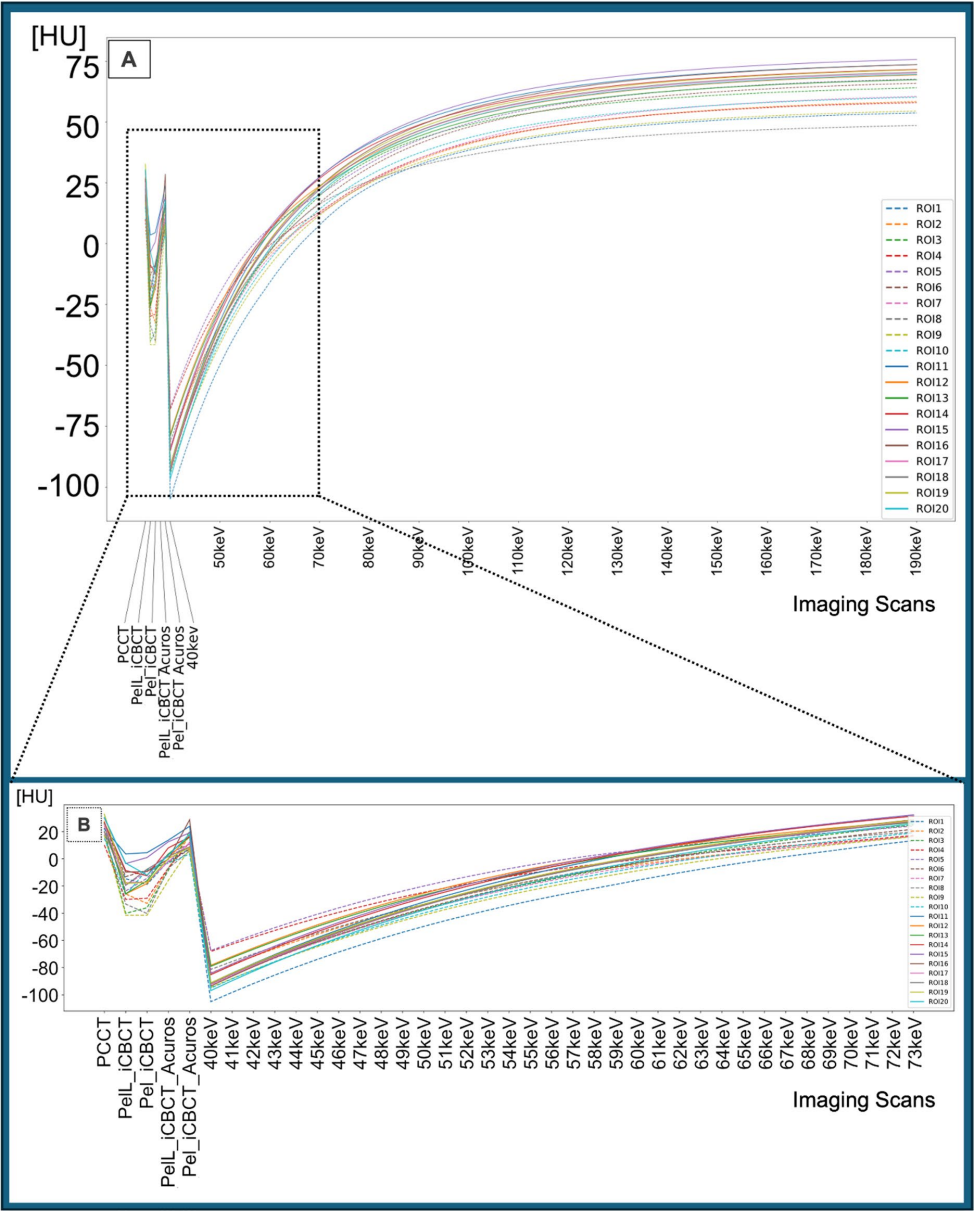
Plot of computed tomography (CT) number measurements in 20 Regions of Interest (ROIs) visualized as ROI-specific curves of a phantom scan with HyperSight conebeam CT (CBCT) with iCBCT and iCBCT Acuros reconstruction modes at two different CBCT presets (Pelvis and Pelvis Large), and photon-counting CT with T3D (PCCT) and virtual monoenergetic reconstructions (40–180 keV) (**A**). A zoomed-in visualization of the CT/CBCT imaging showing only the virtual monoenergetic reconstructions of similar CT numbers (**B**). The corresponding zoomed-in view of section B is marked in section A. The ROIs 1–10 are derived from regions with − 20 HU contrast to background (dashed lines), and the ROIs 11–20 are derived from regions with − 10 HU contrast to background (solid lines).

Nominal CT numbers of the measurement by CBCT and T3D provided comparable values. The VMI at 40–180 keV are characterized by a wide range of increasing CT numbers (Fig. 3).

**Fig. 4:**
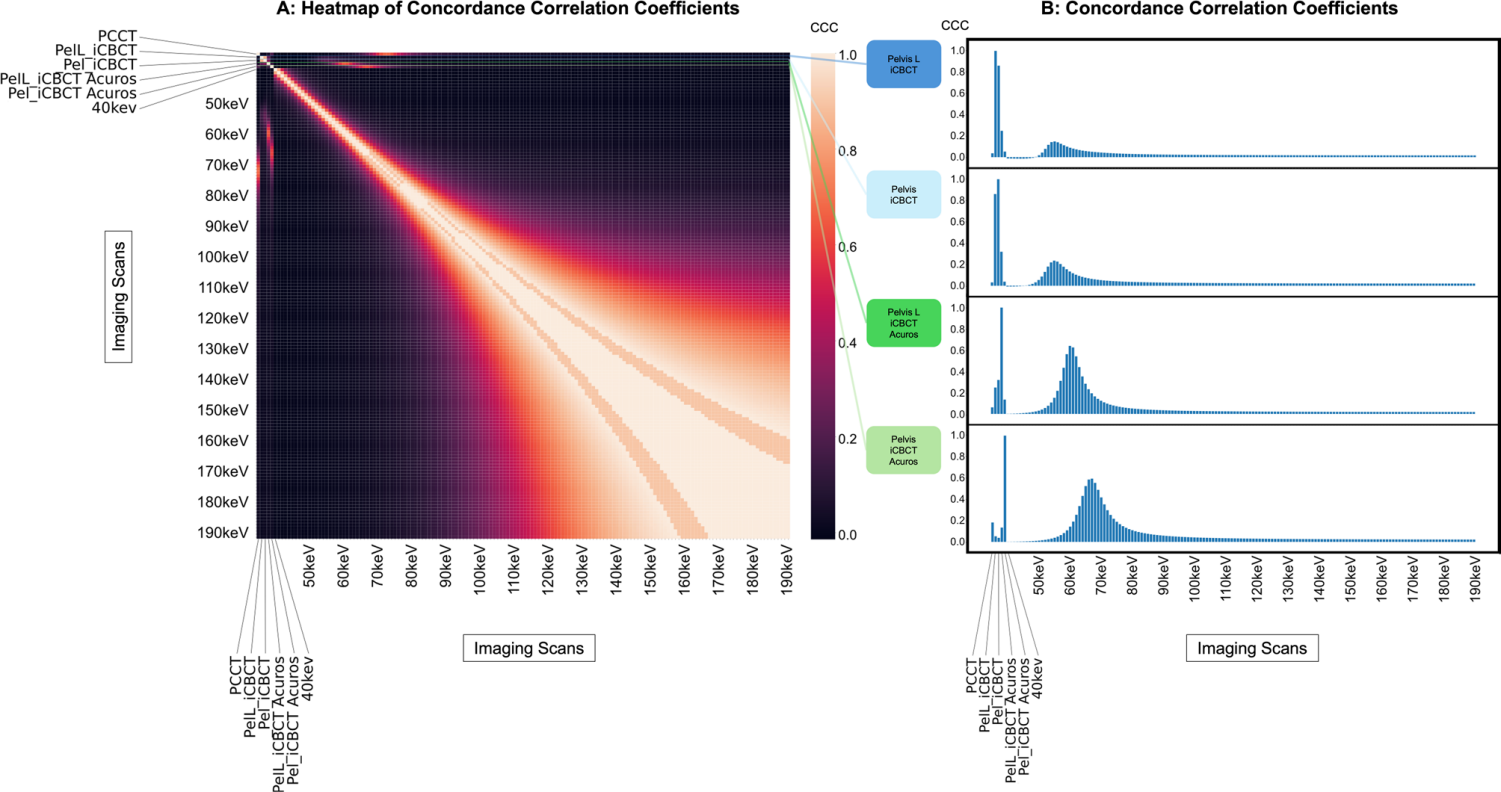
**A** Heatmap of Concordance Correlation Coefficients (CCC) (organized as a symmetric matrix) of the computed tomography (CT) numbers in an inorganic tissue-equivalent anthropomorphic phantom analysis between the HyperSight conebeam CT (CBCT) scan with the presets Pelvis / Pelvis Large (Pelvis L) and the reconstruction modes iCBCT/iCBCT Acuros with both photoncounting CT based T3D (PCCT) and virtual monoenergetic reconstructions (40–180 keV). This heatmap illustrates global concordance patterns. **B**: Plot of the CCCs between the four different CBCT scans and both PCCT-derived T3D and virtual monoenergetic reconstructions (40–180 keV) with the highest values between 55–67 keV. In these selected concordance plots, the x-axis represents the imaging modality, and the y-axis represents the CCC value relative to the CBCT reference imaging mode.

The correlations of CT numbers between T3D and VMIs provided for 3.9% high CCC-values (> 0.5), with the highest peak at VMI 72 keV (CCC = 0.678). The correlations of CT numbers between each of the four different CBCT scans and both T3D and VMIs provided a small range of high CCC between the different measurements (Fig. 4A): 0% for CBCT-Pel_i and -PelL_i, respectively; 2.6% for CBCT-Pel_iA, and 2.6% for CBCT-PelL_iA, respectively.

**Fig. 5:**
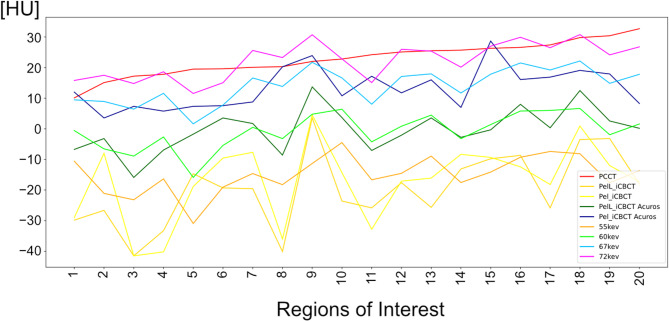
Plot of the computed tomography (CT) numbers of 20 Regions of Interest in an inorganic tissue-equivalent anthropomorphic phantom analysis: Imaging with HyperSight conebeam CT (CBCT) (presets: Pelvis (Pel) and Pelvis Large (PelL); reconstructions: iCBCT and iCBCT Acuros), photon-counting CT based T3D (PCCT), and the virtual monochromatic imaging (VMI) at 55, 60, 67 and 72 keV. Corresponding CT numbers are shown for Pel_iCBCT Acuros with VMI at 67 keV, for PelL_iCBCT Acuros with VMI at 60 keV, and for PCCT-based T3D with VMI at 72 keV. Pel/PelL_iCBCT show partly corresponding CT numbers with VMI at 55 keV.

The CCCs explicitly between the different CBCT scans and both T3D and VMIs demonstrated decreased values with T3D (CCC = 0.031–0.183) and the highest peak with VMI at 55 keV for CBCT_i (CCC = 0.148 and 0.235 for Pel and PelL), at 67 keV for CBCT-Pel_iA (CCC = 0.595), and at 60 keV for CBCT-PelL_iA (CCC = 0.640) (Fig. 4B) with correspondingly similar CT numbers (Fig. 5). The CCCs of CBCT with the other VMIs demonstrated steeply decreasing values at lower and higher energies (Fig. 4B).

The pooled CCCs of the four different CBCT scan modes with T3D and VMIs were significantly different (*p*≤0.001), with increased values both for the reconstruction mode iCBCT Acuros compared to iCBCT (p_adj_≤0.001 and 0.001) and for the preset Pelvis compared to Pelvis Large (p_adj_≤0.001 and 0.001).

### Machine Learning-based validation of the translatability of T3D and VMI to CBCT

**Fig. 6:**
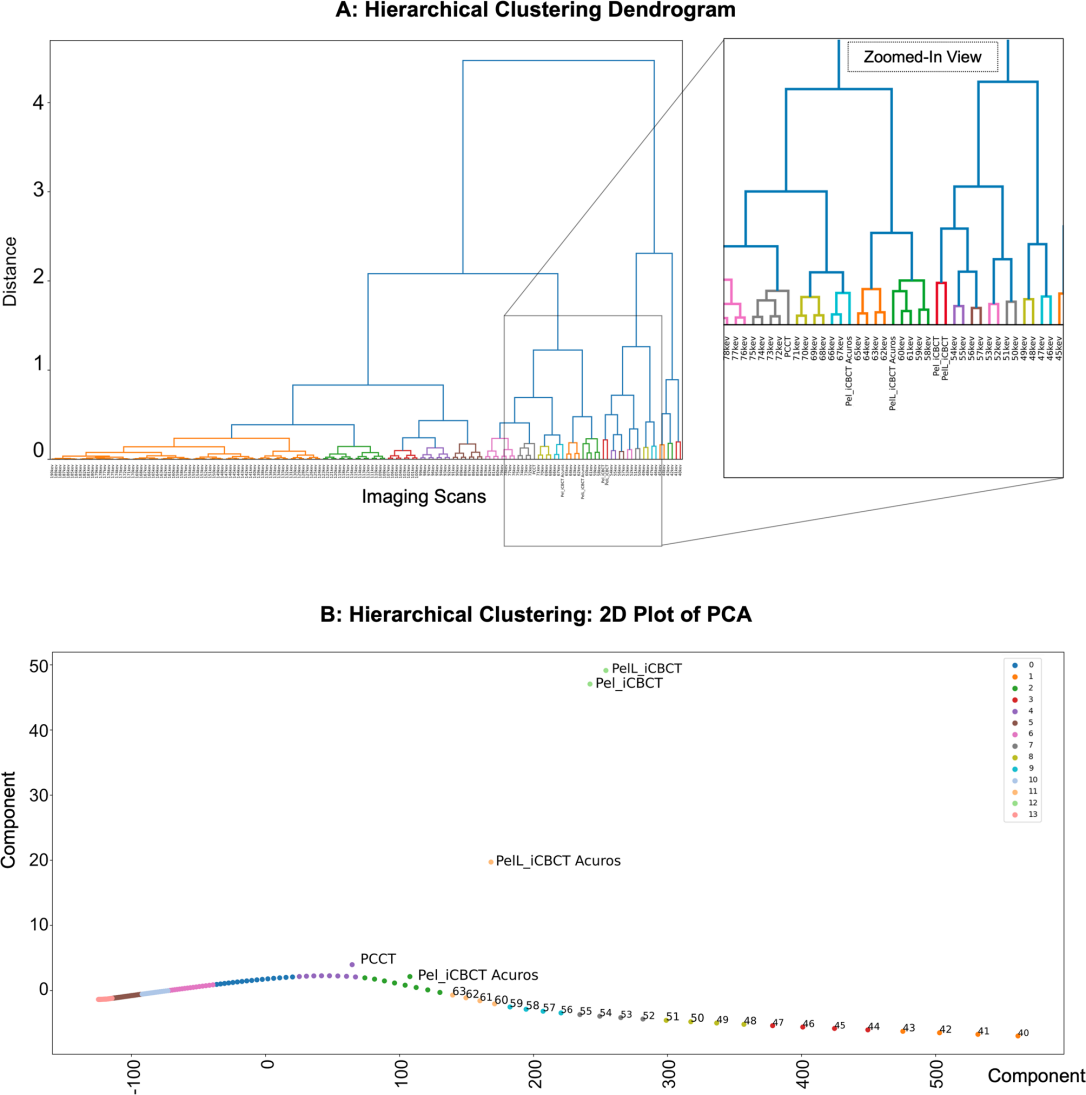
Machine learning-based agglomerative hierarchical clustering approach to investigate similarity across the computed tomography (CT) numbers in an inorganic tissue-equivalent anthropomorphic phantom analysis of 20 regions of interest (ROIs) in different imaging scan modes: Comparison of HyperSight conebeam CT (CBCT) scans (with the presets “Pelvis” (Pel) and “Pelvis Large” (PelL) and the reconstruction modes “iCBCT” and “iCBCT Acuros”) with both photon-counting CT based T3D (PCCT) and virtual monochromatic imaging (VMI) reconstructions at 40–180 keV. **A**: Hierarchical clustering dendrogram with a zoomed-in view of the identified cluster composed of CBCT with distinct VMIs. In the dendrogram, the x-axis corresponds to the imaging scan mode and the y-axis corresponds to the linkage distance, representing the dissimilarity at which clusters merge. The color threshold was established based on the linkage distance corresponding to the last 20 merges, with the aim of highlighting the main clusters. **B**: 2D view of the hierarchical clustering by principal component analysis (PCA) forming 14 different clusters (0–13 with different colours). Based on the PCA algorithm, the first two principal components together captured the majority of the variance and were used as the axes in this figure. Each point in the scatter plot corresponds to a sample projected into the two-dimensional PCA space. The samples were colored according to the hierarchical clustering labels derived from the dendrogram (**A**), which allows for visual inspection of cluster separation in reduced dimensions. PCA based 2D visualization reveals that CBCT imaging forms distinct clusters with VMIs at different keV values. These clusters are separated from T3D PCCT.

By hierarchical clustering of up to 14 clusters with dissimilarity at 0.4864, distinct clusters could be identified consisting of CBCT-Pel_i with CBCT-PelL_i, CBCT-PelL_iA with the VMIs at 58–61 keV, CBCT-Pel_iA with the VMIs at 66–67 keV. T3D was part of a neighboring cluster with the VMIs at 72–75 keV (Fig. 6). This supports the results from the CCC analysis.

## Discussion

This study demonstrates the first comprehensive analysis of qualitative and quantitative, multi-spectral imaging obtained from PCCT scans combined with the novel HyperSight-CBCT scans of high-precision radiotherapy at an Ethos linear accelerator. While the image quality of PCCT imaging was superior to that of CBCT imaging, the iCBCT Acuros reconstruction mode allowed CBCT to partly approach PCCT imaging quality in this phantom analysis.

Most importantly, we could further demonstrate that the correlation between the quantitative imaging parameters of PCCT and those of CBCT imaging is possible by monoenergetic reconstructions and depends generally both on the CBCT preset and on the reconstruction mode. Our study enabled the identification of clusters of highly correlating monoenergetic reconstructions of PCCT matching with specific CBCT scans and significantly exceeding the translatability of polyenergetic CT, thus paving the way for the quantitative translation of multi-spectral PCCT imaging to the novel HyperSight-CBCT imaging in high-precision radiotherapy. As differences in CT value and corresponding quantitative features may be due to different tube voltage values, the advantage of PCCT is the option to choose a matching monoenergetic reconstruction for each CBCT scanning mode.

This research builds upon previous work combining dual-energy CT (DE-CT) and CBCT imaging for radiotherapy. DE-CT imaging improves tissue characterization and expands the amount of information obtained from standard EID-CT^28^. Connecting DE-CT with CBCT improves image quality and may be used to quantitatively translate imaging parameters^29,30^. Preliminary work on implementing DE-CT imaging based on CBCT data may even enable CBCT-based material decomposition^31–33^. Another approach may be to use dual-layer CT imaging for CBCT scans. This application has been demonstrated to be feasible^34^. So, dual-energy imaging shows great promise for future developments of image-guided radiotherapy and may allow for the derivation of spectral information during radiotherapy^35,36^. Additionally, at some point, it may be feasible to develop and clinically establish photon-counting flat panel detectors that could enable dual energy in every CBCT system. Machine learning and artificial intelligence techniques may be essential for modeling the complex and nonlinear relationships between spectral PCCT data and CBCT imaging features, enabling effective mapping across modalities despite differences in hardware and reconstruction algorithms. Such approaches can facilitate the extraction of clinically relevant imaging signatures that may not be directly comparable through conventional methods, thus enhancing the potential for longitudinal tumor monitoring and adaptive radiotherapy.

With the introduction of the novel PCCT technology, CT-based imaging has further evolved significantly from EID-CT and DE-CT into a multi-energy and multi-parametric technique, achieved within a single scan and with potentially reduced dose exposure due to higher dose efficiency^37^. As PCCT imaging not only enhances image quality but also enables the recording of individual photons, it realizes the multi-parametric characterization of the tissues through monoenergetic reconstructions with improved spectral separation compared to DE-CT^38,39^. This capability is particularly advantageous in oncological imaging, allowing for improved characterization of tumor tissues and surrounding normal tissue reactions. Since certain tissues or reactions may be best assessed at specific energies^40^, and as PCCT provides ultra-high resolution imaging that potentially surpasses energy-integrating dual-energy CT imaging regarding image quality^41^ with improving accuracy of target segmentations^42^, PCCT imaging is uniquely suited for daily practice in high-precision radiation oncology.

The multi-parametric characterization enabled by PCCT holds particular importance in oncology, as it aids in tumor characterization and quantification of normal tissue reactions. However, translating PCCT imaging into daily clinical practice of radiation oncology presents challenges. PCCT imaging information, especially ultra-high resolution scan modes, could enhance target delineation precision, for example, in detecting tumor infiltration in bone^43^, while simultaneously reducing dose exposure compared to conventional treatment planning CT scans. This is also critical given the high-dose exposure associated with radiotherapy. This may be even further expanded by the assessment of radiomic features, providing potential markers of oncologic and radio-oncologic imaging^44,45^. However, current data on radiomics in oncologic PCCT imaging, especially in radiation oncology remains limited^46–49^. The integration of these two aspects, namely the assessment of radiomic features extracted from multispectral, high-resolution PCCT imaging rather than from EID-CT, holds significant clinical potential for the development of high-precision radiotherapy. This research domain warrants further exploration in future studies. Despite these promising aspects of synergism, the translation of PCCT imaging to daily IGRT using CBCT remains uncertain, primarily as differences in imaging modalities, such as machine design and reconstruction algorithms, reduce reproducibility in quantitative imaging parameters (e.g. radiomics features)^50^. These challenges may now be mitigated by adopting high-quality CBCT imaging, such as HyperSight-CBCT imaging. The introduction of HyperSight-CBCT imaging represents a significant advancement in radiotherapy imaging, offering improvements in tissue differentiation and target characterization due to enhanced software and hardware, such as larger detector sizes compared to standard CBCT imaging^19–22,51^. So, combining PCCT and its monoenergetic reconstructions with HyperSight-CBCT imaging could offer synergistic value.

Our study demonstrates that it may become feasible to translate multi-spectral information from PCCT to CBCT imaging. However, it is imperative to identify tissue parameters that are either translatable or unique to distinct scan characteristics and thus not transferable. This is crucial for determining the ideal framework and scan characteristics to allow for the optimal connection between PCCT and CBCT imaging. Therefore, future investigations should include the step-wise evaluation of image quality, soft-tissue contrasts, radiomics, and their interaction with ROI identification. This is particularly important when considering the unique characteristics of monochromatic imaging.

Additionally, the high image quality and the quantitative imaging parameters of the novel HyperSight-CBCT mode can be further changed based on the application of different reconstruction modes and scanning presets. In our study, the Pelvis Large preset, which has higher CTDI parameters than the Pelvis preset, provided improved image quality, especially with iCBCT Acuros reconstruction. This aligned it more with PCCT imaging. So, the choice of reconstruction mode and scan preset is a relevant influencing factor for image quality and the optimization of conjunction with multispectral PCCT imaging.

Finally, if realized, the clinical goal could be the identification of a reproducible tumor signature in advanced multispectral PCCT imaging with a corresponding and translatable signature in CBCT imaging, which in turn could enable the monitoring of the relevant tumor volume and allow for image-guided treatment adaptation.

As high-precision radiotherapy combined with HyperSight-CBCT imaging alone already supports the realization of personalized medicine in radiation oncology, especially by means of adaptive radiotherapy, this may be further supported by the integration of advanced imaging modes, as the investigated multi-spectral PCCT. Consequently, this conjunction may enable translational adjustments of ART corresponding to the best matching PCCT based monoenergetic reconstruction. Nevertheless, this highly promising application of ultra-high resolution PCCT imaging in radiation oncology needs to be further investigated in future research^52,53^.

The primary limitation of this study is its phantom design. Since the imaging examinations were conducted using a non-organic phantom, further studies are required to validate these findings in organic ex vivo and in vivo models, especially as HyperSight-CBCT is tuned to human imaging. Additionally, the analysis is valid only for the investigated CBCT presets, meaning that different CBCT presets with alternative scanning parameters may yield varying results. Given that the differences between the CBCT presets Pelvis and Pelvis Large are relatively subtle, the findings may also be caused by other effects than different energy. Another limitation of the study is the comparison of PCCT and CBCT based on CT acquisition parameters that are representative of clinical practice but slightly different, which could influence the translation of CT values.

While CBCT, especially with the Acuros mode, provides higher CT number accuracy than standard CBCT scan modes on linear accelerators, our findings are specific for this high-precision radiotherapy setting. Therefore, future research studies should complementarily investigate the transferability to various imaging techniques frequently used in clinical practice, including different types of linear accelerators (such as the TrueBeam C-arm linear accelerator (Varian Siemens-Healthineers) with partially similar imaging parameters) or devices from other manufacturers. Moreover, the comparison with PCCT was based on standard clinical presets, which utilized different tube voltages, potentially affecting the comparability of the imaging examinations. Further, the size of the phantom combined with fully open collimations, may lead to saturations above and below the phantom, possibly impacting the measured CT numbers. Therefore, the phantom characteristics are of the utmost importance. Future trials should investigate the proposed translational framework of PCCT and CBCT imaging with phantoms of different sizes, as CT numbers and their stability are dependent on the phantom size^54,55^. It is also important to note that increasing phantom sizes may result in scatter increases and CT number degradation^56^. For this reason, the presented phantom investigation, which is based on a large anthropomorphic phantom with an additional fat ring, may be considered a worst-case scenario. At last, the relevance of the phantom shape and its composition is apparent. As demonstrated by the investigated thorax phantom, the bone-like inserts lead to relevant attenuation changes in the posterior part of the phantom. Therefore, it is essential to investigate different anthropomorphic phantoms (e.g., of the pelvis) to address the variability in inorganic, ex vivo and in vivo settings.

These differences could impact CT numbers and the conversion curves for electron density, both of which are critical for dose calculation. Assessing physical density and atomic number, and thus analyzing material decomposition, is an important step in further developing this research field. Consequently, future research should investigate and validate these parameters. Future research should reevaluate the differentiation of distinct CT numbers and, consequently, HU contrasts by the respective imaging scan modes in light of the aforementioned atomic number and physical density. This could lead to the comprehensive and synergistic integration of PCCT imaging for high-precision radiotherapy. Lastly, since this study was conducted at a single institution without external validation, further investigations are necessary to evaluate the reproducibility and performance of high-quality imaging with HyperSight-CBCT, as well as its validation with different PCCT imaging configurations.

In conclusion, this study successfully demonstrates the translation of quantitative CT numbers from multi-spectral PCCT to CBCT imaging by providing specifically matched monoenergetic reconstructions. Based on the conjunction demonstrated between multispectral PCCT and HyperSight-CBCT imaging regarding qualitative tissue visualization and quantitative CT imaging parameters, a baseline has been established for investigating CT imaging signatures of distinct tissues, such as tumor volumes, in both imaging modes. In the future, this may be evaluated in a translational clinical trial with target delineation and treatment planning based on high-resolution, multispectral PCCT imaging and HyperSight-CBCT-based adaptive radiotherapy, followed by multispectral PCCT investigations. If realized, such integration could advance radiotherapy into a more personalized, imaging-based, adaptive treatment approach.

## Data Availability

The data used and generated in this study may be made available, subject to ethical and data protection considerations, upon reasonable request on an individual basis. Please contact Constantin Dreher, MD (E-mail: [constantin.dreher@medma.uni-heidelberg.de]) to request the data.
